# Review on Hyaluronic Acid Functionalized Sulfur and Nitrogen Co-Doped Graphene Quantum Dots Nano Conjugates for Targeting of Specific Type of Cancer

**DOI:** 10.34172/apb.2024.043

**Published:** 2024-03-17

**Authors:** Vinit Sudhakar Patil, Kedar Rupa Bavaskar, Dilip Omprakash Morani, Ashish Suresh Jain

**Affiliations:** ^1^Shri D.D. Vispute College of Pharmacy and Research Center, Devad-Vichumbe, New Panvel, India-410206.; ^2^Department of Pharmaceutics, Shri D.D. Vispute College of Pharmacy and Research Center, Devad-Vichumbe, New Panvel, India-410206.; ^3^Department of Pharmaceutics, Bombay Institute of Pharmacy and Research, Dombivali India-421204.; ^4^Department of Pharmacognosy, Shri D.D. Vispute College of Pharmacy and Research Center, Devad-Vichumbe, New Panvel, India-410206.

**Keywords:** Nanotechnology, Hyaluronic acid, Graphene quantum dots, Cancer, Nanoparticles, Fluorescence, Tumor, Drug delivery

## Abstract

Many people lose their lives to cancer each year. The prevalence of illnesses, metabolic disorders, high-risk infections, and other conditions has been greatly slowed down by expanding scientific research. Chemotherapy and radiation are still the initial lines of treatment for cancer patients, along with surgical removal of tumors. Modifications have been made in chemotherapy since medicines frequently have substantial systemic toxicity and poor pharmacokinetics and still do not reach the tumor site at effective concentrations. Chemotherapy may now be administered more safely and effectively thanks to nanotechnology. Nanotechnology-based graphene quantum dots (GQDs) are very applicable in breast cancer detection, as a drug delivery system, and in the treatment of breast cancer because of their physical and chemical properties, lower toxicity, small size, fluorescence, and effective drug delivery. This paper analyzes the GQDs as cutting-edge platforms for biotechnology and nanomedicine also its application in drug delivery in cancer. It shows that GQDs can be effectively conjugated with hyaluronic acid (HA) to achieve efficient and target-specific delivery.

## Introduction

 Each year, millions of people die from cancer, among the most deadly illnesses.^1^ Because cancer is an aggressive disease, tumor cells frequently spread from primary tumors to faraway organs and tissues. The advancement of metastatic disease indicates a poor prognosis because metastatic illness is typically the ultimate and deadly stage of cancer development.^2^ Despite being the cause of as many as 88% of cancer-related deaths, metastasis is the aspect of cancer pathogenesis that is least understood. During metastatic spread, cancerous cells from a primary tumor follow the processes below: It locally enters the surrounding tissue, move through the lymphatic and vascular systems, survive and largely translocate via the circulatory system for microvessels for distant tissues, leave the bloodstream, endure in the microenvironment of distant tissues, and then ultimately adjusts into the external microenvironment that surrounds these tissues in ways that support the proliferation of cells and the development of a macroscopic secondary tumor.^3^ Breast cancer is the most prevalent malignancy in women and the leading cause of death. Managing breast cancer is becoming more difficult due to metastasis and tumor recurrence.^4^ According to GLOBOCAN statistics, in 2018, there were 18.2 million fresh cases of breast cancer worldwide. Breast cancer is the second major cause of cancer-related death in women, this accounts for 13% of all new cases of cancer and 24% among all cancers in women.^5^ When cells from the tumor mass travel to other areas of the body or infiltrate nearby healthy cells, the tumor mass progresses and turns malignant.^6,7^ Sometimes chemotherapy is started after a mastectomy to entirely eradicate the patient’s metastatic cancer cells.^8^ While chemotherapy successfully extends patients’ lives, anticancer medications have several restrictions on how they can be given to patients. Due to the components’ lipophilicity, oral distribution is severely constrained; therefore, parenteral administration at high concentrations is required to sustain the effective concentration for a prolonged period. There are significant cytotoxic effects brought on by this increased drug concentration in the systemic circulation, including non-specific pharmacokinetics.^9^ Despite the dangers involved, cytotoxic cancer chemotherapy drugs like taxanes and anthracyclines have been an integral treatment component for more than 50 years.^10^ The fusion of engineering and biology is an emerging concept to enhance the delivery of medications directly to tumors and decrease damage to healthy cells and tissue.^11^ To accomplish this, a range of tools and methods have been used, including polymers, lipids, hydrogels, and inorganic carriers.^12^ Moreover, it has been demonstrated that medication resistances have an impact on both the medicines themselves and the bioengineering techniques employed to enhance therapy response.^13^ Hence, utilizing research on human cancer and drug resistance to build tailored nanotherapeutics rationally may help to overcome a lot of these difficulties.^14^ With the help of nanotechnology, it may be possible to increase the solubility and stability of pharmaceuticals as well as their plasma half-lives, reduce side effects that aren’t intended, and concentrate medications where they’re needed.^15^ The selection of viable treatments is based on the features of the tumor, including biomarkers, tumor size, metastatic illness, ligands, antigens, or the expression of endocrine receptors. Chemotherapy and radiation going to be the primary line of therapy for cancer patients, along with surgical resection.^16^ Chemotherapies have been improved since medicines frequently have substantial systemic toxicities and poor pharmacokinetics and still do not reach the tumor site at effective concentrations. Chemotherapy may now be administered more safely and effectively thanks to nanotechnology.^17^ Additionally, nanoparticles are being actively developed for targeted medication delivery, cancer biomarker biomolecular profiling, and in vivo tumor imaging. These nanotechnology-based methods can be used extensively in the treatment of numerous malignant disorders.^18^

## Nanotechnology in cancer targeting

 The administration of medications using nanotechnology can change the path that diseases including cancer, diabetes, infections, neurological disorders, blood-related disease, and orthopedic issues are treated.^19^ For more specific drug targeting, these strategies should ideally improve therapeutic concentration, medication absorption, and stability. An further characteristic of nano-based drug delivery systems is their continuous release of drug within the targeted tissue.^20^ The formation of nano-platforms with certain shapes, surfaces, and size qualities as a consequence of the rational design of nanotherapeutics is essential for biological interactions and the subsequent therapeutic effects.^21^ Formulations that depend on nanotechnology have chemical and physiological properties that are used to treat several illnesses.^22^ The healthcare industry benefits greatly from the current reports on nanoformulations. Most nano-based therapeutic items on the market are intended for intravenous administration, with a few exceptions intended for oral administration.^23^ Numerous preclinical and clinical trials have led to the development of new nanotherapeutics that are not administered through parenteral routes, such as pulmonary, ocular, nasal, vaginal, and cutaneous. For the delivery of drugs, the route of delivery and the associated barriers to be overcome are of particular interest. Many nanoparticle-based formulations have been created over time to enhance the medication delivery mechanism.^24^ Selective cancer targeting has undergone a major transformation thanks to nanotechnology. Nanoparticles may be modified in a variety of ways, including modifying their size, shape, chemical and physical characteristics, and so on, to train them to target certain cells. They can either actively or passively target the cancerous cells. In the case of active targeting, chemotherapeutic agent-containing nano-sized particles are created in a fashion that they interact with the damaged cells directly. Molecular recognition underpins active targeting. As a result, the surface of the nanoparticles is altered to target malignant cells. Targeting agents are often added to the surface of nanoparticles for molecular recognition. Nanoparticles are designed to target malignant cells via ligand-receptor interaction or antibody-antigen recognition.^25-27^

 The three major components of a nanotechnology-based targeted delivery system are

- An apoptosis-inducing agent (anticancer medication).
- A stimulant for targeted moiety penetration.
- A nanoparticle is composed of several different substances.

**Figure 1:**
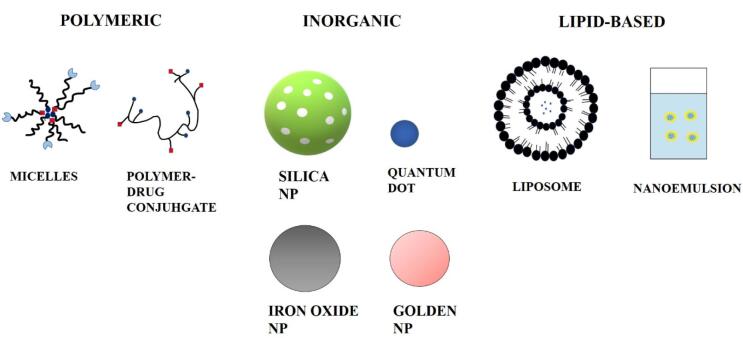
Different Kinds of nanocarriers used for drug delivery

Ceramics, polymers, lipids, and metals are all commonly utilized materials. Cancer can also be targeted by nanoparticles via passive targeting. After apoptosis pauses in cancerous cells, they continue sucking nutritional substances abnormally through the blood vessels, resulting in the formation of broad and leaky blood capillaries around the cells generated by angiogenesis. Basement membrane anomalies and a reduction in the number of pericytes lining rapidly proliferating endothelial cells cause the formation of leaky blood vessels.^28^ Nanocarriers can be classified into three categories based on the materials that they are made from. Different kinds of nanocarriers used for drug delivery are shown in Figure 1.

## Lipid-based nanoparticles

 Most of the lipid-based NPs are spherical platforms with one or more lipid bilayers circulating at the interior aqueous compartment. Lipid-containing NPs comprise several component configurations. Lipid-based NPs have many benefits as a delivery system, such as straightforward formulation, self-assembly, biocompatibility, high bioavailability, capacity for carrying large payloads, and various physicochemical properties that can be controlled to modulate their biological characteristics. These factors make lipid-based NPs the most relevant type of nanomedicines that have received FDA approval.^29,30^

## Polymeric nanoparticles

 Systems of polymeric nanoparticles are created using biodegradable and biocompatible polymers. Dendrimers, polymer-drug conjugates, and micelles are examples of polymeric nanocarriers. Poly lactic-co-glycolic acid (PLGA), poly lactic acid (PLA), and polyethylene glycol (PEG) are just a few examples of the many biodegradable polymers that have been employed to create polymeric nanoparticles.^31^ These nanostructures have also been encapsulated using polysaccharides including pectin, chitosan, and alginate. Block copolymers with variable hydrophilicity and made up of two or more polymer chains are used in the self-assembly method used to create these nanoparticles.^32^ Drugs that are both hydrophilic and hydrophobic can be enclosed in polymeric nanoparticles. This approach permits regulated pH-dependent controlled release and surface changes.^33^

## Inorganic nanoparticles

 Inorganic nanoparticles, which can include iron oxide, silica, gold, and graphene quantum dots (GQDs) among other substances, are one type of nanocarrier that is being created for the detection and treatment of cancer.^34^ These inorganic NPs may be made to have a broad range of sizes, topologies, and geometries since they are carefully formed. The most extensively researched NPs, gold (AuNPs), are employed in a variety of structures, including nanospheres, nanorods, nanostars, nanoshells, and nanocages. Additionally, because of the characteristics of the base material itself, inorganic NPs have special physical, electrical, magnetic, and optical capabilities.^35^ Due to their potential for diagnosis and treatment in anticancer systems, these significant nanoparticles have attracted interest in preclinical investigations. They have a number of applications, including tumour imaging, medication delivery, and advancements in radiotherapy. Recent developments in nanotechnology highlight the significance of inorganic nanoparticles since they can be made of various materials, such as gold, oxide iron, and graphene, and because cells can internalize them through the endocytosis process.^36^

## Types of targeting tumor

 There are two types of tumors that can be targeted: (1) active and (2) passive. In active tumour targeting, biological markers such a natural ligand, an antibody, an aptamer, and a carbohydrate are used to decorate the surfaces of the nanocarriers. Then, through ligand-receptor interaction, the active targeted nano-size drug-carriers (ligand conjugated nanocarriers) will identify and bind to the tumour, and bound nanocarriers are internalized inside the cells.^37^ The enhanced permeability and retention (EPR) effect and defective lymphatic drainage in the tumor’s location, in contrast, are distinct physiological parameters that are dependent on passive tumour targeting. Additionally, passive targeting might help to modify drug distribution following direct tissue delivery.^38^

## Passive targeting

 The purpose of passive targeting is to take advantage of the differences between tumour and normal tissue. Drugs are successfully transported to the target site by passive targeting in order to perform a therapeutic function. Large pores in the vascular wall cause neovascularization, which worsens the permselectivity of tumour vessels relative to healthy vessels.^39^ High cancer cell proliferation also promotes neovascularization. Macromolecules, such as NPs, can leak from blood vessels supplying the tumour and accumulate within tumour tissue as a result of the fast and deficient angiogenesis.^40^ The retention of NPs is increased in cancer due to poor lymphatic drainage, which enables the nanocarriers to transfer their contents to tumour cells. One of the driving forces for passive targeting, the EPR effect, is brought on by these processes. The tumour microenvironment, in addition to the EPR effect, plays a significant role in the passive distribution of nanomedicines.^41^

## Active targeting

 In order to extend circulation time and achieve passive targeting coupling of a particular ligand on the surface that will be recognized by the cells present at the illness site, active targeting to the disease site relies on addition to PEG modification of nanocarriers.^42^ The therapeutic drug must be conjugated to a ligand that is specific to a tissue or cell in order to accomplish active targeting.^43^ To transport drugs to the target region, various types of nanoparticles have been produced. These ligands are unique in that they have the ability to identify and attach to complimentary molecules, or receptors, that are present on the surface of tumour cells. A greater amount of the anticancer medicine locates and enters the tumour cell when such targeting molecules are introduced to drug delivery nanoparticles, boosting treatment effectiveness and lowering harmful effects on surrounding healthy tissue.^44^

## Hyaluronic acid

 Hyaluronic acid (HA) commonly known as hyaluronan is an anionic and nonsulfated glycosaminoglycan that is found widely in connective and epithelial tissues Mucopolysaccharide, is the most crucial part of the extracellular matrix.^45^ helps significantly in cell proliferation and migration and therefore is responsible for the growth of several malignant tumors.^46^ HA, a nonsulfated linear glycosaminoglycan found mostly in the extracellular matrix, regulates tissue hydration through its high hydrophilicity and water-holding capacity.^47^ Because of its superior biocompatibility, selective targeting, and high drug-loading capacity, it is a promising biopolymer for bioconjugation.^48^

 HA can be used as a tumor site-specific drug delivery method due to its high binding affinity for the CD44 receptor, a member of the cell adhesion protein family, which is overexpressed along the outermost layers of numerous carcinoma cells, including breast cancer cells.^49^ CD44, on the other hand, has been demonstrated to be expressed at very low levels on normal As a result, HA-modified nanoparticles or micelles appear to be viable carriers for CD44-targeted chemotherapy agents.^50^ HA has multiple functional groups that are employed in various conjugations and modifications. Because of these qualities, HA is an important component of multipurpose NPs used to administer synergistic cancer therapy.^51^ To make use of HA’s targeting characteristics, many ways of generating HA NP formulations have evolved.^52^

## Quantum dots

 One of the terms for quantum dots (QDs) is ‘artificial atoms,’ because they have distinct energy levels and their bandgap may be accurately adjusted by adjusting their size.^53^ They may be a great source of light ranging from UV to IR based on their size and composition. A QDs crystal core has around 100-100 000 atoms.^54^ QDs are semiconductor crystals with nano-sized scales made up of elements from groups II to VI or III to V, and they are described as particles having physical dimensions less than the exciton Bohr radius.^55^ A quantum dot generally has a diameter of 2 to 10 nm. The diameter of the QD depends on the chemicals used in synthesis.^56^

 QDs have distinct luminescence or electronic features, like broad and sustained narrow emission, absorption spectra, and good photostability.^57^ These absorb white light and, based on the band gap of the material, release an identifiable color a few nanoseconds later.^58,59^ QDs made with different legends or anticancer agents/genes to concurrently image tumor cells, and malignant growth treatment via particular authority to receptors overexpressed on tumor cells and tissue surface may substantially improve fluorescent bioimaging and target delivery proficiency.^60^

## Graphene quantum dots

 The scientific community has been interested in carbon nanoparticles because of their optical, thermal, electrical, and mechanical properties. They have a range of functionalization chemistry and are safer and more secure than metal-based nanoparticles for usage in cancer theranostics.^61,62^ GQDs have garnered a lot of interest since carbon is among the most prevalent compound on the planet and because they may sometimes take the place of semiconducting QDs in certain applications.^63^

 Scientists have recently paid a lot of attention to GQDs, which show exciton confinement and the quantum-size effect and are composed of very thin (usually 3-20 nm) graphene sheets. The scientific community has been interested in carbon nanomaterials because of their distinctive electrical, optical, thermal, and mechanical capabilities. For use in cancer theranostics, they have a variety of functionalization chemistry and are safer and more secure than metal-based nanoparticles^64,65^ Since carbon is one of the most common elements on Earth and because they might occasionally replace semiconducting QDs in some applications, GQDs have attracted a lot of attention.^66^

 Recently, scientists have focused a lot of interest on GQDs, which are made of incredibly thin (about 3–20 nm) graphene sheets and exhibit confinement and the quantum-size effect. GQDs also exhibit tunable luminescence, photobleach resistance, constant Photoluminescence (PL), and high solubility in a variety of solvents. Colloidal inorganic semi-conductive QDs, which are poisonous due to the leakage of heavy metals including cadmium, and zinc from their core and have gained a lot of interest in coming years for their electrical and optical properties.^67^ GQDs effectively take the place of coating. GQDs are separate from carbon nanodots (C-dots), even though they are classed as the same thing. C-dots are NPs with PL properties that are quasi-spherical and have a diameter of less than 10 nm. GQDs, on the other hand, are graphene nanosheets containing functional groups (carboxyl, carbonyl, hydroxyl, and epoxide) commonly located at their edges. They normally have a thickness of less than 10 nm and a lateral dimension of 100 nm. By changing their electron density, these groups can operate as reaction sites and affect PL emission from the QD.^68^

## Synthesis of GQDs

 The known approaches for the synthesis of GQDs may be broadly classified as top-down and bottom-up procedures. As with bottom-up approaches, the synthesis of GQDs needs complicated reaction pathways and particular organic ingredients, making optimization challenging. Therefore, it is preferable to employ the top-down strategy, which entails fragmenting big blocks of carbon material.^69^ There are several top-down strategies for the synthesis of GQDs, including chemical oxidation methods,^70-72^ hydrothermal methods,^73,74^ ultrasonic aided methods,^75^ electrochemical oxidation methods,^76^ chemical vapor deposition methods,^77-79^ and pulsed laser ablation (PLA) techniques,^80-83^ GQDs feature a hexagonal crystalline structure of carbon atoms grouped in rings of six atoms, which is a graphene lattice by definition, even if the structure of GQDs depends on the synthesis circumstances.^84^

## Different methods of synthesis of QDs

 Different methods of synthesis of GQDs are given in following Table 1.^85-87^

**Table 1:** Different methods of synthesis of graphene quantum dots

**Type**	**Method**	**Advantages**	**Disadvantages**
Top down	Oxidation method	Most used method for large-scale production since it is easy to use and efficient.	Strong oxidizers that must be utilized may burn or explode
	Hydrothermal/ solvothermal method	It is an easy and quick procedure. Hydrothermal technology is also ecologically beneficial.	Long reaction time, a reaction also involves high pressure and temperature.
	Electrochemical oxidation	high levels of stability and a homogeneous size distribution	Product yield is poor.
	Microwave-assisted/ ultrasonic-assisted process	It can increase the manufacturing yield in addition to reducing response time.	Costly ultrasonic reactor.
Bottom up	Controllable method	GQDs gets homogeneous shape and size	Multistep complex process and low quantum yield
	Carbonization	It is an easy and ecologically beneficial technique.	tough to properly control

## Structure of GQDs

**Figure 2:**
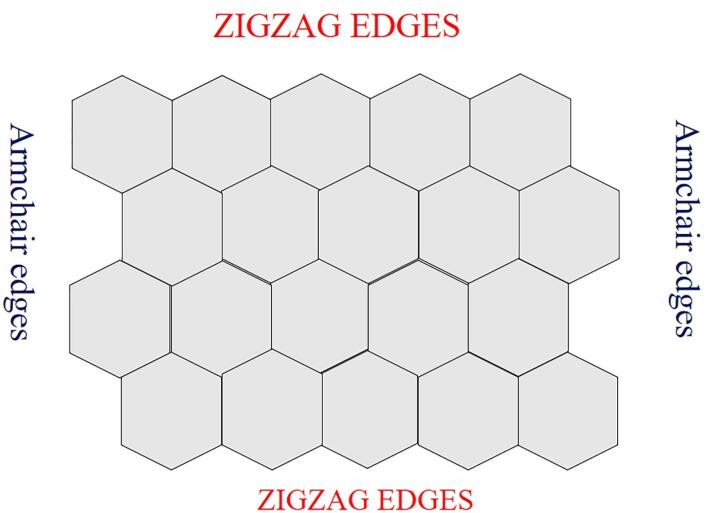
Structure of GQD

 Each carbon atom is covalently linked to three more carbon atoms in this way, resulting in the sp^2^ hybridized property to the sheet’s plane. These characteristics are responsible for their exceptional electrical and optical qualities.^88,89^ GQDs have two sorts of edges: armchair and zigzag. Triple carbon bonds may be seen at the armchair edges, and the edge has a carbene-like structure. Carbene-like edges are defined by the presence of two unshared valence electrons on each carbon atom at the zigzag edges.^90-92^ The edge types influence the form of GQDs and, as a result, their optical and electrical characteristics.^93^ Structure of GQD is shown in Figure 2.

## Photoluminescence mechanism of GQDs

 GQDs are the most basic Carbon dots, having a single-layer carbon core and chemical groups attached on the surface or edges. Therefore, GQDs are an excellent model for studying the PL methodology of CDs. The PL behavior of chemically produced GO is provided first to explain the PL mechanism of GQDs since GO is an essential raw material for GQD synthesis and consequently GO and GQDs have comparable chemical structures. Oxygen-based functional groups are found in GO, around the edges, or on the basal plane. As a consequence, a linearly aligned epoxy and hydroxyl-boned sp^3^ C-O matrix cover the 2-3 nm aromatic sp^2^ domains.^94,95^ Fluorescence can be facilitated by radiation-induced recombination of electron-hole (e-h) pairs in such sp^2^ clusters.^96^ The band gaps of various sp^2^ size distributions encompass a wide range due to the large size distribution of sp^2^ domains in GO, resulting in a broad PL emission spectrum from visible to near-infrared. Many researchers have looked at the fluorescence of GO and decreased GO.^97^ GQDs are more porous, have more surface functional groups, and have more surface flaws than GO. The GO fluorescence measurements can be utilized to help explain these emissions in GQDs. Graphene excitants have an unlimited Bohr diameter. As a result, any size graphene fragment will exhibit quantum confinement effects. GQDs have a non-zero band gap and PL on excitation as a result. By altering the GQDs’ size and surface chemistry, the band gap may be changed. In the recent five years, there has been a significant advancement in the production of GQDs, and researchers have identified plausible PL mechanisms: conjugated surface/edge state π-domains.^98^

## Bioimaging with GQDs

 Nowadays, non-toxic diagnostic imaging for cells has been created. When compared to other materials, GQDs show a substantial advantage in cell imaging.^99,100^ With the use of different wavelengths of the electromagnetic spectrum, bioimaging is a significant technology that is employed in both research and clinical contexts. It enables the monitoring of biological processes such as targeted delivery, cellular uptake, and biodistribution of medicines in a precise, isolated manner.^101-103^ Imaging is important in cancer diagnosis because sensitive imaging allows quick cancer diagnosis in addition to the recognition of metastatic or the recurrence of the disease.^104^ Based on modified GQDs, targeted tumor cell imaging can be achieved. This is possible when GQDs are changed or connected with certain bioactive species. Sun et al. employed chemically abridged GQDs and photo-reduced GQDs as fluorescent probes to image A549 cells in a typical investigation.^105^

## Functionalization of graphene quantum dots

###  Doped graphene quantum dots

**Figure 3:**
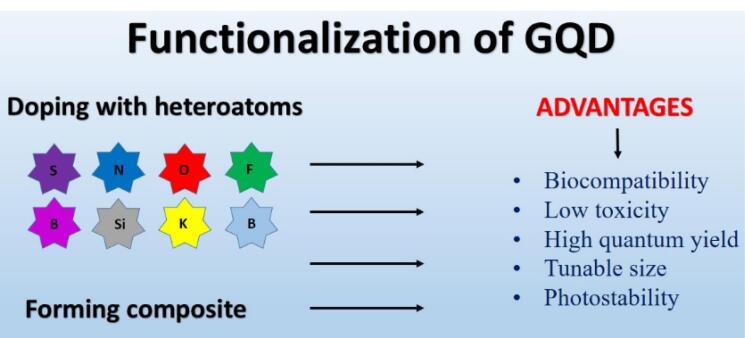
Advantages of functionalization of GQDs

 Together with transition metals and metal atoms like Si and Mn, the bulk of the atoms doped with GQDs include N, C, P, B, and S. Metals and transition metal ion ions can be hazardous, restricting their usage in fluorescence sensing applications where toxicity is a concern. Also, due to the larger radiuses of metal and transition metal atoms than those of carbon, doping with GQDs frequently ends in dispersed and inefficient integration. As a result, doping with nonmetallic atoms offers clear advantages. GQDs doped with nonmetallic atoms are more biocompatible for use in human medicine. Additionally, because non-metal ions and carbon atoms have comparable sizes, they may be included uniformly and modify the electronic structure of GQDs, causing physical flaws that enhance the QY and particular binding properties of GQDs.^106^ Figure 3 shows the advantages of Functionalization of GQDs.

## Nitrogen doping (N-GQDs)

 It is feasible to create nitrogen doped GQDs via the constant inclusion of nitrogen since nitrogen has a comparable size to carbon and may establish strong covalent connections with it.^107^ The electrical characteristics and surface state defects of GQDs are enhanced by N-doping, and the amount of reactive groups enhances the fluorescence quantum yield of GQDs. Because of the recent interest in N-GQDs, a range of methods for producing N-GQDs have been developed. The preparation begins with the combination of a carbon and nitrogen source, followed by heating in a poly tetrafluoroethylene-lined tank, followed by separation, and then purification. Transmission electron microscopy (TEM), atomic force microscopy (AFM), and X-ray diffraction are regularly used to clarify structure, whereas X-ray photoelectron spectroscopy (XPS), and infrared spectroscopy (IR spectroscopy) are usually used to establish chemistry (X-ray diffraction (XRD)). Citric acid is a popular source of carbon that has been coupled with ethylenediamine, glutamic acid, urea, ammonia, or glycine as a nitrogen source. The use of glycine in the manufacture of N-GQDs can result in consistent size distributions (e.g. 2.2 1.5 nm), with fluorescence excitation and emission wavelengths of 353 nm and 450 nm, respectively.^108^ N-GQDs were synthesized by Du et al using GO and ammonia. TEM yielded an average diameter of 5 nm. The resulting N-GQDs glow bright blue when exposed to UV light.^109^

## Sulfur doping and Co-doping

 Sulfur may also be utilized to dope GQDs, resulting in different characteristics. The size of the s atom exceeds that of the carbon atom. Because the outermost orbitals of two atoms differ, In GQDs, the combination of S leads to an unequal spin density distribution.^110^ The electronegativity distinction between S and C is quite modest, indicating that there is little driving force for electron transport between the two materials.^111^ All of these traits make it challenging to integrate S doping into a GQD architecture. Despite these obstacles, many techniques for synthesizing S-doped GQDs (S-GQDs) have recently been published. Functionally, bottom-up process steps were similar to the techniques discussed in the previous section for manufacturing N-GQDs and require a carbon and a sulfur source.^112^ Citric acid, 3-mercapto-succinic acid, and NaOH were employed as synthetic ingredients to create S-GQDs in DMF. Bian et al. used 1,3,6-nitropyrene as the carbon source, with either Na2S or 3-mercaptopropionic acid (MPA) as the sulfur atom source. In AFM, the synthesized S-GQDs exhibit a homogeneous particle size distribution when utilizing MPA, with an average diameter of 2.5 nm and an average height of 0.8 nm. XPS indicates the presence of carbon, sulfur, and oxygen on the surface of S-GQDs, as well as carboxyl groups. A solution of S-GQDs looks yellow and generates intense blue fluorescence when exposed to 365 nm ultraviolet light. The fluorescence intensity rises from 310 nm to 360 nm, then decreases when the excitation wavelength goes higher from 375 nm to 390 nm. These S-GQDs have maximal excitation and emission wavelengths of 360 nm and 450 nm, respectively.^113^ After synthesizing S-GQDS with citric acid and MPA as raw materials, Kadian et al used FT-IR and XPS to demonstrate the C-S bond. The peak excitation and emission wavelengths of S-GQDS were measured to be 340 nm and 440 nm, respectively. It indicates that the sulfur atom was effectively doped into GQDs.^114^

## Doping with other heteroatoms

 There have been reports of doping of GQDs with additional atoms, including K, Si, B, Cl, S, and P, in addition to the individual and combined doping of N and S. Doping changes the properties of GQDs. The BC bond is 0.7% longer than the CC bond, and electron loss causes energy state defects in GQDs, resulting in surface defect discharge. Boron-GQDs (B-GQDs) can thereby alter the optical characteristics of GQDs by generating a high number of active sites.^115^ Ge et al synthesized B-GQDs using a NaOH solution utilizing 1,3, 6-nitropyrene as a source of carbon as well as borax as a source of boron. B-GQDs produce a brilliant yellow solution. The B-GQDs showed good crystallinity as evidenced by TEM; AFM demonstrated graphene having one or two layers of thickness; and XPS analysis of the components revealed that the B-GQDs have abundant groups that contain oxygen on their surfaces. XPS also demonstrated that B atoms were successfully integrated within the GQD lattice. In visible light, the solution that forms of B-GQDs is pale yellow, while in ultraviolet light, it is green.^116^

## GQDs based drug delivery systems

 Prior to the advent of nanotechnology, organic fluorescent dyes were thought to be useful tools for bioimaging applications. The previously held idea, however, has been altered by recently released luminous nanomaterial kinds.^117,118^ In order to achieve targeted and visible medication administration, QD, which are important in nanomedicine, enable the integration of medicines, affinity ligands, and imaging moieties within a single nanostructure. These semiconductor nanoparticles help transport various kinds of efficient anti-cancer medications for gene therapy and immunotherapy in addition to improving the pharmacologic features of currently used treatments. In overall, nanoparticle-based drug delivery methods improve circulation times, minimise drug toxicity, increase bioavailability, and regulate drug release and targeting. Because of this, drug delivery that utilizes nanocarriers has a number of benefits over traditional drug delivery systems.^119^

## Recently used quantum dots to target tumors

 Different types of QD have been used recently for targeting cancer cells or tumors, such as nitrogen-doped GQDs, graphitic carbon nitride QDs, black phosphorus QDs, etc.^120-126^ A list of these QD and their activities is given in Table 2.

**Table 2:** List of quantum dots used for tumor targeting

**Quantum dots**	**Activity**	**Reference**
Nitrogen-doped GQDs	Spread of cancer cells	^121^
MnO2 QD stabilized by cysteine	Dopamine detection	^122^
pH-responsive black phosphorus QDs	Photodynamic treatment for tumors	^123^
Graphitic carbon nitride QDs	Combination chemo-photodynamic treatment for tumors	^124^
Black phosphorus QDs	Synergistic chemo-phototherapy and dual-modality cancer imaging	^125^
CQDs - quinic acid	Gemcitabine to breast cancer cells	^126^
Duplex metal co-doped CQDs	Synergistic cancer treatment	^127^

## GQDS-HA release remedy

 As demonstrated by XPS, dopamine hydrochloride conjugated to HA has been attached to a GQD that takes the fascinating adhesive properties of the catechol molecule. Transmission electron microscopy confirmed the particle size as being 20 nm, and the spectrum of fluorescence revealed significant fluorescence power despite HA attachment.

**Figure 4:**
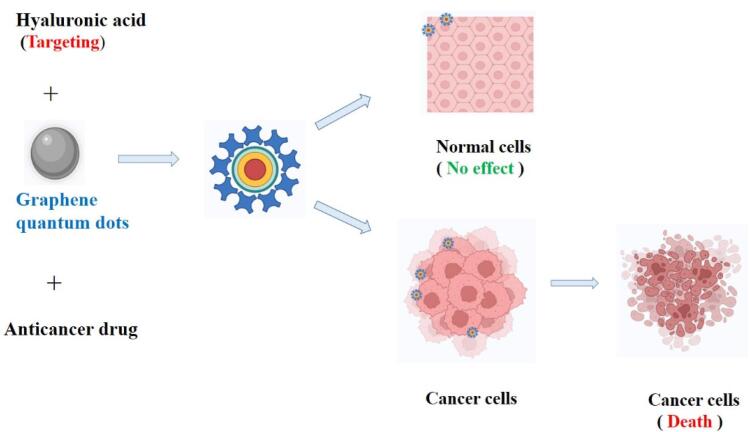
HA-GQD coupling and targeting to cancer cell

 Al-Nahain et al investigated how to use hyaluronic acid (GQD-HA) as a targeting agent to deliver a GQD efficiently and precisely to the intended target. A GQD that adopts the intriguing binding capabilities of dopamine which is the catechol moiety, has been used to attach HA. In vivo, biodistribution analysis showed that the tumor tissue emitted more intense fluorescence when the produced GQD-HA was administered to female Balb/c mice with tumor-bearing CD44 receptors. Using a confocal laser scanning microscope, in vitro cellular imaging revealed that CD44 overexpressed A549 cells fluoresced brightly. Results from both in vitro and in vivo experiments demonstrated the value of utilizing HA as a targeting moiety. The hydrophobic drug doxorubicin’s loading and release kinetics from a GQD in moderately acidic circumstances demonstrated that a GQD may be thought of as a new drug transporter, and the MTT assay’s nontoxic results support the claim that GQD-HA is a biocompatible substance.^101^ HA-GQD coupling and targeting to cancer cell is shown in Figure 4.

 Wu et al studied the HA conjugation to graphene oxide for targeted drug delivery. HA binds to nanoscale graphene oxide through the creation of amide bonds after adipic acid dihydrazide functionalizes GO to add amine groups. The outcomes of the toxicological tests conducted in vivo and in vitro demonstrate that the resultant GO-HA displays minimal cytotoxicity, great blood compatibility, and no overt toxic effect on mice. Experiments on cellular uptake show that the GO-HA can specifically carry anticancer medicines into the cells through receptor-mediated endocytosis. The anticancer medication DOX has a high loading capacity for the GO-HA, and the resultant GO-HA/DOX demonstrates a high level of cytotoxicity to HeLa cells.^127^

 Zheng et al investigated the detection of human tumor cells using HA-linked nitrogen-doped GQDs (N-GQDs). Blue luminous N-GQDs were created utilizing a hydrothermal method approach that was speedy and easy to prevent by-products, highlighted the binding sites to ensure correct arrangement, and were simple to utilize. In an outcome of the nitrogen component doping, an amide II bond is adequately established, and several binding sites for HA crosslinking were provided. MCF-7 cells fluoresced strongly when CD44, which was overexpressed on the surface of the cells, was combined with HA-conjugated N-GQDs (HA-N-GQDs). HA-N-GQDs’ excellent fluorescence, low toxicity, and high cytocompatibility suggested that they may be used in fluorescence imaging enabling accurate detection of cancer cells.^128^

## Conclusion

 Recent advances in GQD research have demonstrated GQDs’ potential as novel platforms in biotechnology and nanomedicine. Novel experimental techniques were developed to enhance the physicochemical characteristics of GQDs to ensure they adhere to the requirements of a specific application. Thus, incorporating the receptor-binding molecule into the GQD can improve cell efficiency. Because of its biocompatible, biodegradable, and nontoxic properties, HA is widely used as a primary receptor, it also has a high affinity for the CD44 receptor. Catechol, the side chain of the rare amino acid 3,4-dihydroxy-L-phenylalanine, L-DOPA, often known as DN, is a common component of marine mussel adhesion substances is notable as a bonding agent for surface modification and biological systems. As a result, HA-DN nanotherapeutics are an excellent cancer treatment alternative.

## Acknowledgments

 All individuals listed as authors have contributed substantially to the work and are required to indicate their specification contribution.

## Competing Interests

 Authors declare no conflict of interest.

## Ethical Approval

 Not applicable.
